# Assessment of Natural Killer Cell Serial Degranulation

**DOI:** 10.1002/cpz1.70452

**Published:** 2026-08-24

**Authors:** Jens A. Niemann, Carsten Watzl

**Affiliations:** ^1^ Department of Immunology Leibniz Research Centre for Working Environment and Human Factors (IfADo) Dortmund Germany

**Keywords:** cytotoxicity, degranulation, natural killer cells, serial killing

## Abstract

Natural killer (NK) cells have been shown to be heterogeneous and to differ in their ability to kill target cells: although some NK cells show little cytotoxic activity, others sequentially kill multiple targets. The latter are called serial killers, and they perform this activity mainly via the release of cytotoxic granules. During this degranulation process, CD107a is exposed at the NK cell's surface. By staining this extracellular CD107a in a sequential manner, the ability of NK cells to serially degranulate while in co‐culture with target cells can be analyzed. Here we describe a protocol for the detection and characterization of serial degranulating human NK cells. © 2026 The Author(s). Current Protocols published by Wiley Periodicals LLC.

**Basic Protocol**: Natural killer cell serial degranulation assay

## INTRODUCTION

Human NK cells are able to sequentially kill multiple target cells in a process termed serial killing (Bhat & Watzl, 2007). Although only a fraction of NK cells engage in serial killing, it enables a minority of the NK cells to perform the majority of the cytotoxic function (Srpan et al., 2018; Vanherberghen et al., 2013). This makes serial killing NK cells interesting for therapeutic applications in which the cells’ cytotoxic activity is important. Bulk cytotoxicity assays such as chromium or calcein release assays do not provide any information about how many NK cells are active and how active they are; they are just a readout of the average cytotoxic activity of the cell population. To analyze serial killing on a single‐cell level, live‐cell time‐lapse microscopy can be used, but these approaches are time consuming and limited in throughput. The serial degranulation assay described here eliminates these problems by relying on flow cytometry and is thereby also easily combined with phenotyping of the cells (Niemann et al., 2025). However, additional information about NK cell‐to‐target cell contact dynamics and NK cell motility that is obtained via imaging is lost with this flow cytometry‐based approach.

NK cells use two distinct pathways to induce target cell death (Mace et al., 2014). The lytic granule‐mediated pathway predominates in serial killing (Prager et al., 2019). To kill a target cell, the NK cell releases the effector molecules perforin and granzyme from their lytic granules onto the target cell, a process called degranulation. During degranulation, the lytic granules fuse with the NK cell plasma membrane to release their content. Via this fusion process, integral membrane proteins of the granules gain access to the plasma membrane. One such protein is CD107a, which is commonly used as marker of degranulation (Alter et al., 2004). Through staining of CD107a multiple times, the assay gives a readout about when and how often an individual NK cell degranulated during co‐culture with target cells. However, it is important to note that although degranulation correlates with killing, it does not directly measure the death of target cells but rather the ability of an NK cell to be activated multiple times. Therefore, serial degranulation is only a proxy for serial killing NK cells because it is not certain that each degranulation event leads to a kill.

This article provides a basic protocol for measuring the serial degranulation of human NK cells. It uses peripheral blood mononuclear cells (PBMCs) as effector cells and K562 as target cells. Other cells present within the PBMCs do not interfere with or modulate the natural cytotoxicity of the NK cells in this assay (Niemann et al., 2025). This basic protocol includes information on how to stain, wash, and co‐incubate the cells. The discussion of variations in the Background Information section below provides possible alterations or extensions to the main protocol that have already been tested.

## NATURAL KILLER CELL SERIAL DEGRANULATION ASSAY

The serial degranulation assay aims to assess the ability of NK cells to degranulate multiple times in a sequential manner when in co‐culture with target cells. This is achieved by using the same clone of an anti‐CD107a antibody linked with different fluorophores. After each labeling step, unlabeled externalized CD107a is blocked with a non‐conjugated anti‐CD107a blocking antibody, after which the antibodies are removed by a washing step, and the effector cells are given time to degranulate again before the next fluorophore‐labeled antibody is added. When the assay is conducted properly, the NK cells can be identified as having degranulated between zero and three times.

The addition of the blocking antibody significantly reduces the false positive labeling of an early degranulated NK cell during a later labeling step (Niemann et al., 2025). To keep the results comparable between repeats of the experiment, the timing of the steps needs to be identical (see figure in Time Considerations, below). Therefore, the overall sample size should be no more than 12, so that all samples may to be handled in parallel with a multichannel pipet.

**Figure 1:**
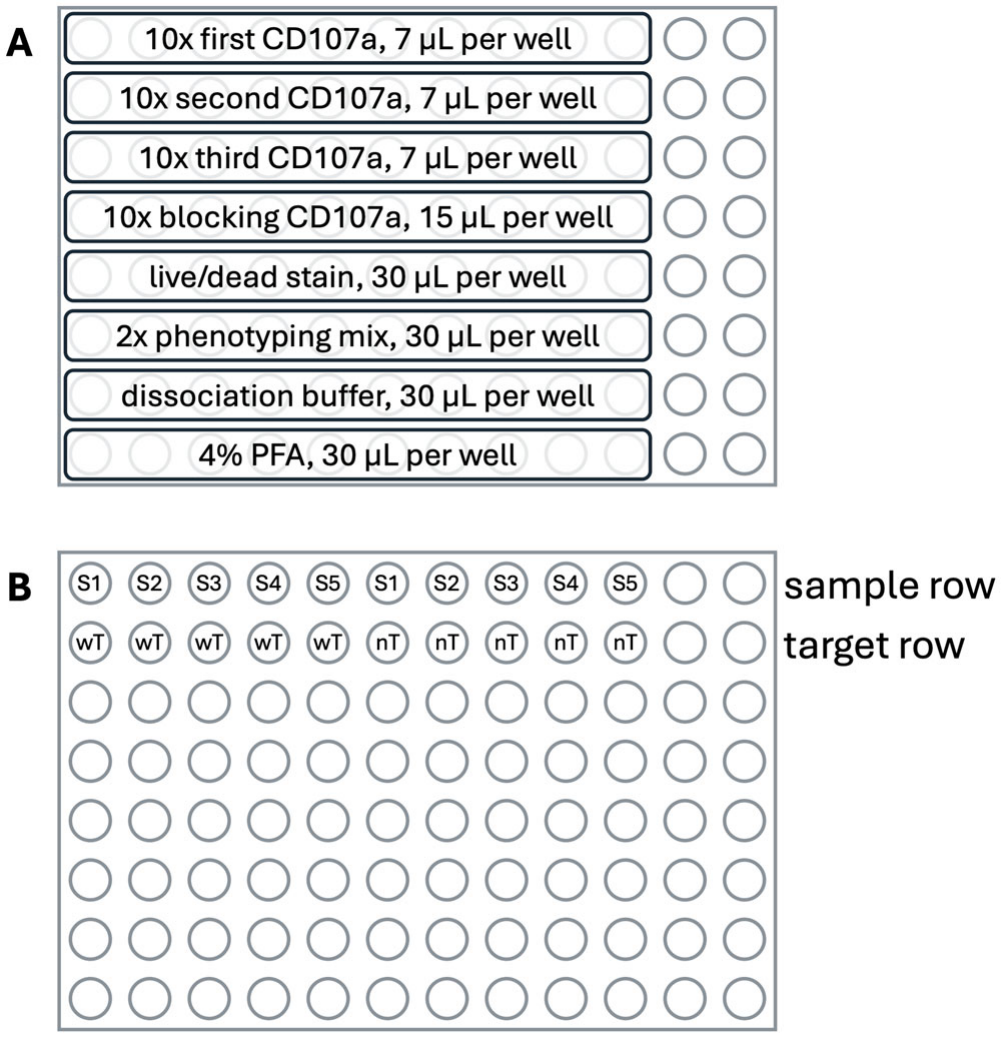
Example plate layouts. (**A**) Example layout of the reagent plate for 10 samples. (**B**) Example layout of the sample plate for five conditions (S1‐S5] resulting in 10 samples (5 samples with target cells [wT] and 5 without target cells [nT]).

For each sample with target cells, it is necessary to also set up a corresponding sample without target cells to ensure that the assay itself does not stimulate the NK cells. During the assay, samples should be kept in the sample plate at 37°C, and reagents should be kept on the reagent plate on ice. Examples of sample and reagent plates are shown in Figure 1.

### Materials

PBMCs, rested overnight after isolation or thawing, at 5 million cells/ml in IMDM GlutaMAX (Thermo Fisher Scientific, cat. no. 31980030) + 10% FBS (Thermo Fisher Scientific, cat. no. 10270106); if low activity is expected, 0.5 ng/ml IL‐15 can be added during the resting period (but note that this will pre‐stimulate CD56^bright^ NK cells more strongly than CD56^dim^ cells; Wagner et al., 2017)
K562 cells (ATCC, cat. no. CCL‐243)
Assay medium (see recipe)
10× anti‐CD107a‐BV421 (see recipe)
10× anti‐CD107a blocking antibody (see recipe)
10× anti‐CD107a‐FITC (see recipe)
10× anti‐CD107a‐APC (see recipe)
Live/dead stain (see recipe)
2× phenotyping mix (see recipe)
Dulbecco's phosphate‐buffered saline (DPBS) without calcium and magnesium (Thermo Fisher Scientific, cat. no. 14190169)
Fetal bovine serum (FBS; Thermo Fisher Scientific, cat. no. 10270106)
Enzyme Free Cell Dissociation Solution (Merck KGaA, cat. no. S‐014‐C)
4% (w/v) paraformaldehyde (PFA) solution (see recipe)

96‐well V‐bottom plates (Thermo Fisher Scientific, cat. no. 249570) and corresponding lids
200‐ and 50‐µl multichannel pipets
Centrifuge with inlet for 96‐well plates
Incubator, 37°C, 5% CO_2_
Dry heating bath (able to keep a 96‐well plate at 37°C)
Ice bucket
Flow cytometer (Cytek Aurora or equivalent, able to detect at least six different fluorescence signals)

1 Prepare a 96‐well V‐bottom plate (reagent plate) with the necessary staining and blocking solutions as shown in Figure 1A.
  To avoid air bubbles, prepare 7 µl to pipet 5 µl and 30 µl to pipet 25 µl
2 For each sample, add 1 million effector cells (PBMCs) to one well of a V‐bottom plate (sample plate).
3 For each sample for which target cells (K562 cells) are needed, add 1.1 million cells to the well below (target cell row, on the sample plate; see Fig. 1).
4 Spin down for 5 min at 500 × *g*, 20°C, and remove supernatant.
5 Add 55 µl assay medium for each sample to the target row, and also to wells with no target cells.
  Addition of assay medium should be done from a reservoir to allow use of a multichannel pipet.
6 Resuspend and mix cells by pipetting.
7 Transfer 50 µl from the target row onto the sample row with a multichannel pipet.
  This ensure that all samples come into contact with target cells simultaneously.
8 Resuspend and mix cells by pipetting.
9 Spin down for 10 s, 20°C, to enable direct contact formation.
  Most centrifuges have a “quick spin” function that accelerates as long as you hold down the button; do this for 10 s or until 300 g is reached.
10 Incubate the plate for 10 min at 37°C, 5% CO_2_.
11 Add 5 µl of 10× anti‐CD107a‐BV421 and resuspend the cells by pipetting them up and down to separate cell‐cell contacts.
  Each time small volumes are added (such as 5 µl), the volume for pipetting up and down should be increased. Pipetting a volume of 30 µl up and down ensures proper resuspension without the risk of introducing air bubbles.
12 Incubate the plate for 5 min at 37°C, 5% CO_2_.
  To avid opening the incubator too much, alternatives such a dark box in a heating bath can be used if HEPES is included in the assay medium. This applies to all the 5‐min incubation steps.
13 Add 5 µl of 10× anti‐CD107a blocking antibody and resuspend the cells by pipetting them up and down.
14 Incubate the plate for 5 min at 37°C, 5% CO_2_.
15 Wash by adding 100 µl assay medium and centrifuging for 5 min at 500 × *g*, 20°C.
16 Remove supernatant.
  Flicking out the supernatant ensures fast and uniform removal.
17 Resuspend in 50 µl assay medium and mix cells by pipetting.
18 Spin down for 10 s, 20°C, to enable direct contact formation.
19 Incubate the plate for 10 min at 37°C, 5% CO_2_.
20 Add 5 µl of 10× anti‐CD107a‐FITC and resuspend the cells by pipetting up and down to separate cell‐cell contacts.
21 Incubate the plate for 5 min at 37°C, 5% CO_2_.
22 Add 5 µl of 10× anti‐CD107a blocking antibody and resuspend the cells by pipetting them up and down.
23 Incubate the plate for 5 min at 37°C, 5% CO_2_.
24 Wash by adding 100 µl assay medium and centrifuging for 5 min at 500 × *g*, 20°C.
25 Remove supernatant.
26 Resuspend in 50 µl assay medium and mix cells by pipetting.
27 Spin down for 10 s, 20°C, to enable direct contact formation.
28 Incubate the plate for 10 min at 37°C, 5% CO_2_.
29 Add 5 µl of 10× anti‐CD107a‐APC and resuspend the cells by pipetting them up and down to separate cell‐cell contacts.
30 Incubate the plate for 5 min at 37°C, 5% CO_2_.
31 Wash by adding 100 µl assay medium and centrifuging for 5 min at 500 × *g*, 4°C.
32 Remove supernatant.
33 Add 25 µl live/dead stain and resuspend the cells by pipetting.
34 Incubate the plate on ice for 10 min in the dark.
35 Add 25 µl of 2× phenotyping mix and resuspend the cells by pipetting them up and down.
36 Incubate the plate on ice for 15 min in the dark.
37 Wash by adding 100 µl of DPBS + 2% FBS and centrifuging for 5 min at 500 × *g*, 4°C.
38 Remove supernatant.
39 Resuspend cells vigorously in 25 µl Enzyme Free Cell Dissociation Solution.
40 Incubate the plate on ice for 10 min.
41 Pipet up and down again to separate cell‐cell contacts.
42 To fix the cells, add 25 µl of 4% PFA and mix by pipetting them up and down.
43 Incubate the plate on ice for 10 min.
44 Wash by adding 100 µl DPBS + 2% FBS and centrifuge for 5 min at 500 × *g*, 4°C.
45 Remove supernatant.
46 Resuspend cells in 150 µl DPBS + 2% FBS.
47 Analyze samples by flow cytometry.
  An example of a gating scheme is provided in Understanding Results, below.

## REAGENTS AND SOLUTIONS

Final concentrations mentioned are those in the assay—that is, after the predilution has been added to the sample. A predilution of 10× that gives a concentration of 1 µg/ml in the assay would itself have a concentration of 10 µg/ml. The volumes listed are examples for an experiment with 5 conditions and therefore 10 samples (one sample with and one without target cells for each condition).

**Table:** 

***Assay medium***	
Iscove's modified Dulbecco's medium (IMDM; Thermo Fisher Scientific, cat. no. 12440053)	7120 µl
10% FBS (Thermo Fisher Scientific, cat. no. 10270106)	800 µl
1 M HEPES (final concentration 10 mM; Thermo Fisher Scientific, cat. no. 15630080)	80 µl
***Anti‐CD107a‐BV421, 10×***	
Assay medium (see above)	68 µl
Anti‐CD107a‐Brilliant Violet 421 (BV421; 1:5; final concentration 1 µg/ml; clone H4A3, BioLegend, cat. no. 328625)	17 µl
***Anti‐CD107a‐FITC, 10×***	
Assay medium (see above)	80.25 µl
Anti‐CD107a‐fluorescein isothiocyanate (FITC; 1:20; final concentration 1 µg/ml; clone H4A3, BioLegend, cat. no. 328605)	4.25 µl
***Anti‐CD107a‐APC, 10×***	
Assay medium (see above)	80.25 µl
Anti‐CD107a‐allophycocyanin (APC; 1:20; final concentration 1 µg/ml; clone H4A3, BioLegend, cat. no. 328619)	4.25 µl
***Anti‐CD107a blocking antibody, 10×***	
Assay medium (see above)	140 µl
Anti‐CD107a, unconjugated (1:5; final concentration 10 µg/ml; clone H4A3, BioLegend, cat. no. 328601)	35 µl
***Live/dead stain***	
DPBS (Thermo Fisher Scientific, cat. no. 14190169)	349.5 µl
Zombie NIR Fixable Viability Kit (BioLegend, cat. no. 423105; 1:700 dilution)	0.5 µL
***Phenotyping mix, 2×***	
DPBS (Thermo Fisher Scientific, cat. no. 14190169)	338.1 µl
2% FBS (Thermo Fisher Scientific, cat. no. 10270106)	7.0 µl
Anti‐CD3 BUV563 (1:100; final concentration 1 µg/ml; clone UCHT1, Waters Biosciences, cat. no. 569789)	3.5 µl
Anti‐CD56 BUV805 (1:250; final concentration 0.4 µg/ml; clone B159, Waters Biosciences, cat. no. 742022)	1.4 µl
***Paraformaldehyde (PFA), 4% (w/v) (200 ml)***	
Distilled H_2_O, heated to 60°C	180 ml
Paraformaldehyde (Merck, cat. no. 16005 1KG R)	8 g
2 N NaOH	2 drops
Let mixture stand until clear (after a few minutes) and then remove from heat	
10× DPBS (Thermo Fisher Scientific, cat. no. 14200075)	20 ml
HCl (adjust pH to 7.2)	
Store up to 1 year at –20°C	

## COMMENTARY

### Background Information

The serial degranulation assay offers a platform that can be expanded and adjusted depending on your research question.

#### Effector cells

The protocol described here uses whole PBMCs and gates on NK cells in the analysis. The assay can also be performed with isolated NK cells. In that case, the cell ratios need to be adjusted to keep the effector‐to‐target ratio (E:T) at ∼1:10. If cell numbers become too high, a smaller E:T can be chosen, but the target cells need to outnumber the NK cells at least threefold, or even more if high degranulation is expected.

NK cells can also be cultured and stimulated with cytokines before the assay to analyze the influence of these conditions on serial degranulation.

#### Target cells

Target cells other than K562 can be used. Adherent cells are also suitable if they are detached before the assay (note that some detachment solutions, such as trypsin, might change the expression of ligands for NK cell receptors on the target cells).

#### Incubation times

Target cells that activate NK cells to a lesser degree than K562 cells might necessitate an increase in incubation time to obtain sufficient degranulation. However, co‐incubation times should not exceed 30 min as this can result in loss of serial degranulation resolution. This is because NK cells degranulating multiple times during one incubation period are only stained for one degranulation event. Thereby, long incubation times, which will allow more opportunity for multiple degranulation events of individual NK cells, may result in data actually showing a reduced amount of triple degranulation. Information about contact dynamics between the NK cell and the target cell, as obtained by time‐lapse microscopy, can be helpful to determine whether a timeframe is too long and could enable NK cells to degranulate multiple times. Please note that the time between contact to degranulation is shorter than the time to target cell death and that NK cells do not need to search around for new targets in the serial degranulation assay.

#### Phenotyping

Dependent on the capabilities of the flow cytometer used to measure the samples, the phenotyping panel can be expanded to compare serial degranulation capabilities of NK cell subpopulations.

#### CD107a staining

It is possible to switch out the fluorophore‐labeled anti‐CD107a antibodies for different ones. When choosing other fluorophores, it is important to check that all anti‐CD107a antibodies used in the assay produce the same positive population. To validate this, co‐incubate your effector and target cells and then stain them with all three anti‐CD107a antibodies simultaneously. Each antibody should result in the same percentage of positive effector cells. Preferably, this is achieved by using the same amount of antibody. If it is necessary to switch to a different anti‐CD107a clone, all antibodies need to be switched, as well as the blocking antibody.

#### Analysis

Instead of directly analyzing the cells with a flow cytometer, fluorescence‐activated cell sorting can be used to isolate NK cells showing different degranulation capabilities and use them for further experiments: e.g., continued cultivation, microscopy, or RNA sequencing.

### Critical Parameters

To keep the result comparable between experiments, it is critical that the timing of the steps be the same between experiments. Also, pipetting with the multichannel pipet should be practiced so that the cells can be resuspended quickly and without producing air bubbles. Because timing is very important, samples should be prepared in a single row of a 96‐well plate, if possible, so that they can be all pipetted into simultaneously with a multichannel pipet.

The V‐well plate leads to quite compact cell pellets. Make sure each time you resuspend the cells that the pellet is completely gone. NK cells need to be separated for each staining and blocking step. After the serial degranulation part of the protocol is done, keep the cells on ice to prevent further NK activation. To preserve the fluorophores, they and the labeled cells should be kept in the dark when possible.

### Troubleshooting

Problems, their possible causes, and applicable solutions for the serial degranulation assay can be found in Table 1.

**Table 1:** Troubleshooting for Serial Degranulation Assay

Problem	Possible cause	Solution
Low overall degranulation	NK cells do not get enough activating signal from interacting with the target cells	Increase co‐incubation time between CD107a stainings

### Understanding Results

**Figure 2:**
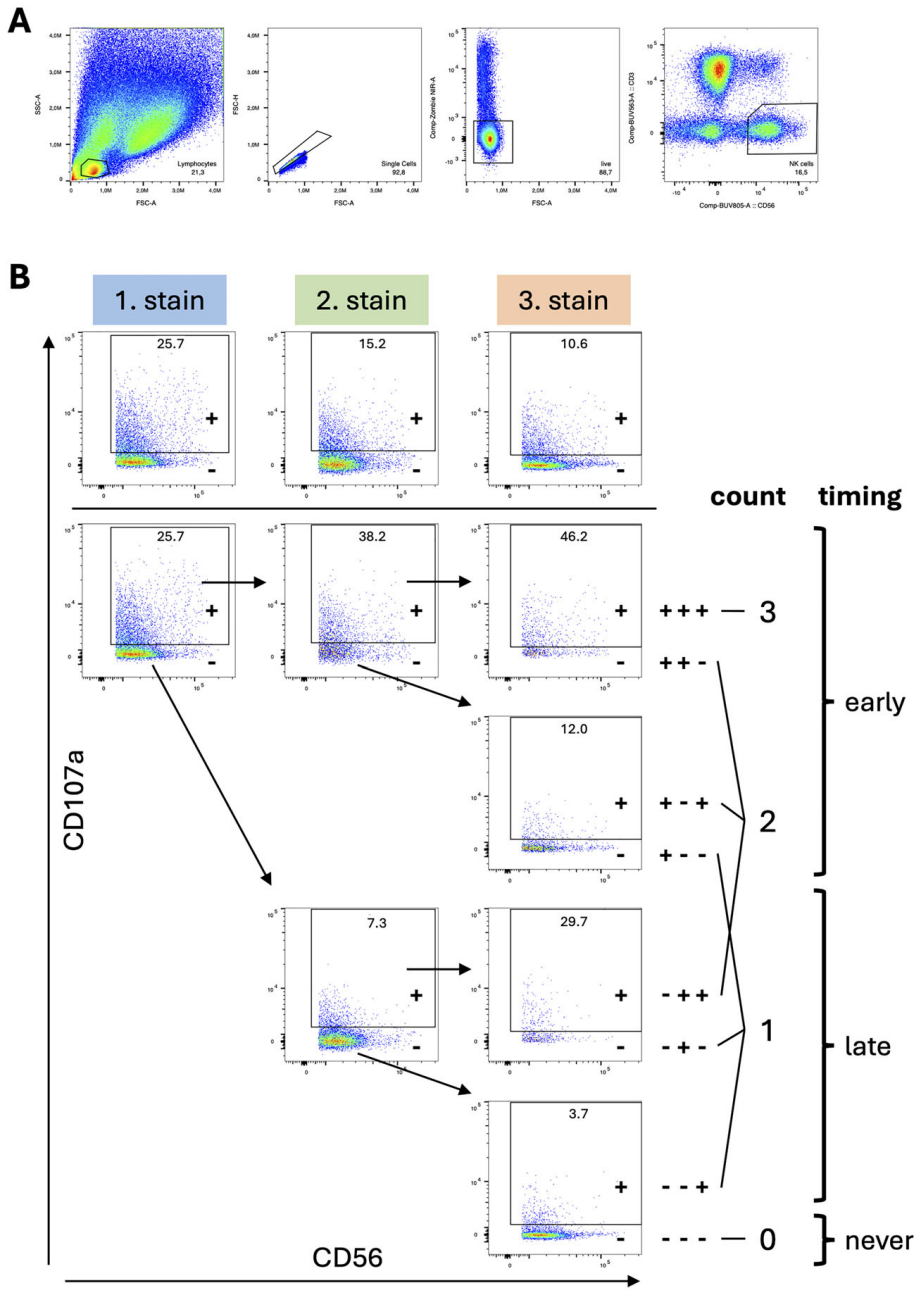
General gating schemes. (**A**) NK cells were gated according to size, as single cells, negative for the dead cell dye Zombie NIR and for CD3 negative and positive for CD56. (**B**) Gating strategy of CD107a‐positive NK cells from total NK cells (top) or nested gating according to a decision tree (bottom) for eight different degranulation outcomes and combined according to count of degranulations or degranulation timing.

**Figure 3:**
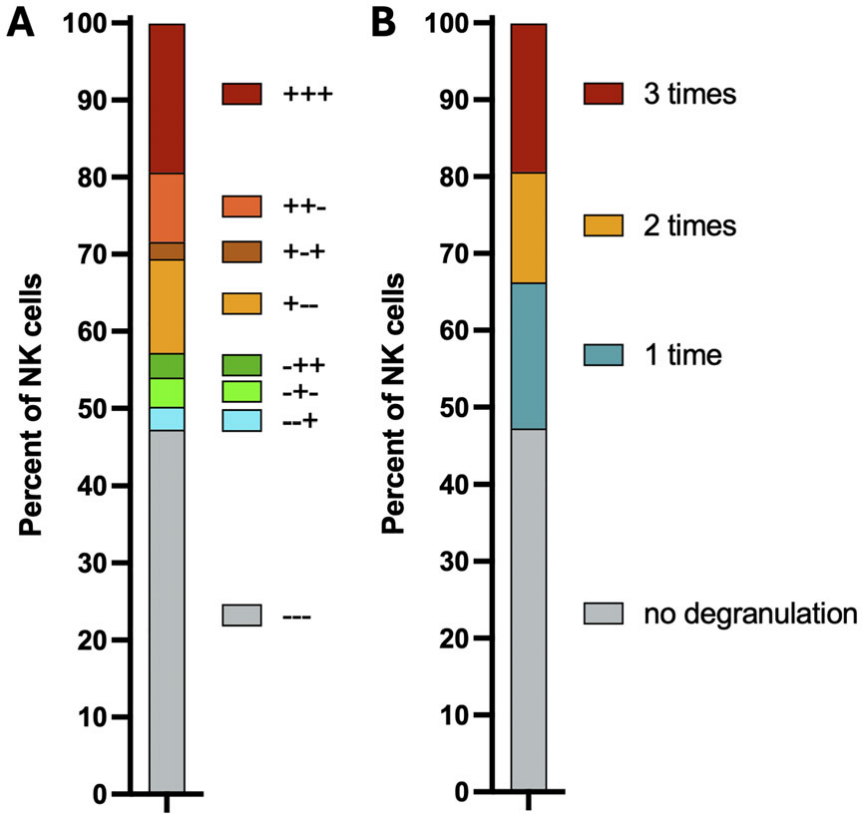
Visualization of serial degranulation outcome. (**A**) Example of the visualization of serial degranulation data showing all different degranulation groups. (**B**) Example of the visualization of serial degranulation data combined according to count of degranulations.

The first step in the analysis is to gate on the NK cells. An example is shown in Figure 2A, where NK cells are gated according to size, as single cells that are negative for the dead cell dye Zombie NIR and for CD3 and positive for CD56. From there, the CD107a gates are set according to unstimulated NK cells. For each CD107a‐positive gate, the corresponding not‐gate is created. Then, a nested gating scheme is set up as shown in Figure 2B. Depending on whether and when an NK cell degranulates, it will end up in one of eight degranulation groups. Denoting CD107a‐positive, degranulated NK cells with a “+” and ones that did not degranulate with a “‐” for each of the three stainings, the following degranulation outcomes are possible: +++, ++‐, +‐+, +–, ‐++, ‐+‐, –+, and —. These can be combined again based on the count or timing of the degranulation (Fig. 2B). Figure 3 gives examples of how to visualize all eight degranulation groups (Fig. 3A) and how to visualize them combined according to the number of degranulations (Fig. 3B).

When using FlowJo to analyze the data, it is possible for rounding of the results to lead to degranulation groups that do not add up to 100%. To avoid this, it is advisable to use FlowJo to deliver the cell counts and use another program to calculate the fractions from the counts.

### Time Considerations

**Figure 4:**
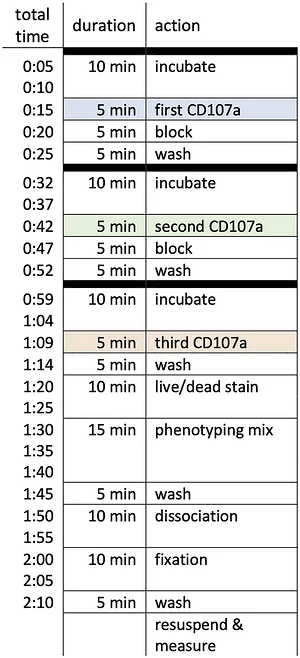
Assay timeline. Timeline of multiple degranulation protocol. Black bars indicate short spin‐downs and are allocated 2 min. Total time (hours:minutes) starts after the first short spin‐down.

The duration of the serial degranulation part of the assay, as demonstrated here for K562 target cells, is fixed and will always be 70 min, as shown in the timeline in Figure 4 (although other target cells may require different co‐incubation times). The phenotyping afterwards takes ∼60 min (other, more involved phenotyping approaches may take longer). Measuring time varies dependent on the number of samples. When performing the assay for the first time, the preparation of cells and staining solutions can take about an hour. Once one is comfortable with the assay, this preparation time can be shortened by preparing necessary staining solutions while the cells are already co‐incubating (this is only advised if one can guarantee that the preparation will be done in time and this will not affect the duration of the incubation and staining steps). Depending on number of samples and familiarity with the assay, the entire assay will take between 3 and 4 hr.

### Author Contributions

**Jens A. Niemann**: Methodology; investigation; writing—original draft; formal analysis; visualization. **Carsten Watzl**: Conceptualization; funding acquisition; writing—review and editing; project administration; supervision; resources.

### Conflict of Interest

The authors declare no conflict of interest.

## Data Availability

The data, tools, and material (or their sources) that support the protocol are available from the corresponding author upon reasonable request.
